# Real-world comparison of variable vs fixed-loop circular pulsed field ablation catheters: Acute outcomes including non-pulmonary vein ablation

**DOI:** 10.1016/j.hroo.2025.08.030

**Published:** 2025-08-21

**Authors:** Joerg Yogarajah, Julie Hutter, Patrick Kahle, Marko Tomic, Mirlinda Lüsebrink, Andreas Hain, Samuel Sossalla, Malte Kuniss, Thomas Neumann

**Affiliations:** 1 Department of Cardiology, Kerckhoff Heart Center, Campus Kerckhoff, Justus Liebig University Giessen, Bad Nauheim, Germany, and; 2 Department of Cardiology, Medical Clinic I, Justus Liebig University Giessen, Giessen, Germany

**Keywords:** Atrial fibrillation, Pulsed field ablation, Catheter ablation, Atrial flutter, Cavotricuspid isthmus ablation, Left atrial roof ablation, Posterior wall isolation

## Abstract

**Background:**

Pulsed field ablation (PFA) is an emerging non-thermal approach for pulmonary vein isolation (PVI) in atrial fibrillation (AF). Comparative real-world data between variable-loop circular catheter (VLCC; VARIPULSE™, Biosense Webster, Inc) and fixed-loop circular catheter (FLCC; PulseSelect™, Medtronic) catheters, including non-PVI ablation, are limited.

**Objective:**

To compare acute efficacy, procedural characteristics, and safety of PVI, and adjunctive ablations performed with VLCC vs FLCC during clinical implementation.

**Methods:**

Overall, 90 consecutive patients were studied (first 45 VLCC and 45 FLCC cases). FLCC procedures used fluoroscopic guidance; VLCC was integrated with 3-dimensional mapping, reflecting standard real-world use of each catheter. Additional ablations were performed at the operator’s discretion. Acute outcomes and complications were analyzed.

**Results:**

Acute PVI success was 100% in both groups. Additional ablations (cavotricuspid isthmus [CTI], roof lines, posterior wall, superior vena cava [SVC]) were performed in 17 (VLCC) and 15 (FLCC) patients, achieving bidirectional block without Radiofrequency touch-ups. In PVI-only cases, FLCC was associated with shorter median procedure time (67.2 vs 76 min, *P* < .001), whereas VLCC had reduced fluoroscopy time (8.3 vs 11.4 min, *P* < .001). Major complication rates were low and comparable (2.2% vs 0%, *P* = 1).

**Conclusion:**

This first clinical comparison demonstrated high acute efficacy and favorable safety profiles, with notable procedural differences reflecting their distinct workflows of 2 circular PFA catheter systems for AF ablation. Likewise, this includes the first reported successful CTI, SVC and mitral isthmus ablation using VLCC reflecting its versatility for ablation. Further research is warranted to assess long-term outcomes and lesion durability.

**Key Findings**

▪ This is the first real-world comparison of two circular pulsed field ablation catheter systems: a variable loop circular catheter (VLCC; VARIPULSE™, Biosense Webster) and a fixed loop circular catheter (FLCC; PulseSelect™, Medtronic) during early clinical adoption.
▪ Both systems achieved 100% acute pulmonary vein isolation success with low complication rates; FLCC was associated with shorter procedure times, whereas VLCC required less fluoroscopy.
▪ Both VLCC and FLCC enabled successful non-pulmonary vein ablations (cavotricuspid isthmus, roof line, posterior wall isolation, mitral isthmus, superior vena cava) without the need for radiofrequency touch-up.
▪ Feasibility of VLCC use in redo atrial fibrillation ablation procedures has been demonstrated.

## Introduction

Pulsed field ablation (PFA) has emerged as a major innovation in the interventional treatment of atrial fibrillation (AF), enabling non-thermal, tissue-selective lesion formation with a favorable safety profile compared to conventional thermal energy sources.^1^ While PV isolation (PVI) remains the cornerstone of AF ablation, there is growing interest in the use of PFA for substrate modification beyond the PVs, including linear lesions such as the left atrial (LA) roof line or the cavotricuspid isthmus (CTI).

Among emerging single-shot PFA platforms, the FARAPULSE™ system (Boston Scientific) with its pentaspline design has seen the most widespread adoption.^1^^,^^2^ Recently, however, 2 additional circular, multipolar PFA catheter systems have entered clinical use: PulseSelect™ (Medtronic; fixed-loop circular catheter [FLCC]) and VARIPULSE™ (Biosense Webster, Inc; variable-loop circular catheter [VLCC]). Pre-market studies and pivotal trials for both systems have shown promising outcomes.^3^, ^4^, ^5^ Despite both being circular multipolar systems, they differ substantially in design and workflow integration: the FLCC is operated under fluoroscopic guidance without electroanatomic mapping, whereas the VLCC is fully integrated into 3-dimensional (3D) mapping systems and features a variable-loop design. These fundamental differences may affect procedural duration, fluoroscopy exposure, overall workflow efficiency, and versatility for non-PV ablation.

Although some real-world data support the safety and efficacy of FLCC for PVI, evidence on its performance for non-PV ablations remains limited.^6^ Likewise, real-world experience with the VLCC is scarce, especially regarding ablation targets beyond the PVs, such as CTI or the superior vena cava (SVC).^7^ To date, no head-to-head real-world data exist comparing these 2 circular PFA systems in terms of procedural characteristics, acute efficacy, and the feasibility of PVI or extended ablation strategies during their early clinical implementation.

This study addresses this gap by providing the first real-world comparison of VLCC and FLCC systems. Special emphasis is placed on acute outcomes and the feasibility of additional ablations beyond PVI including roof lines, CTI, and SVC ablations, performed during the initial adoption phase of each technology.

## Methods

### Study population

This observational study analyzed the first 90 consecutive patients with symptomatic AF who underwent initial PFA at a high-volume electrophysiology center during the respective clinical implementation phases of 2 PFA systems. The first consecutive 45 patients treated with VLCC between March and May 2025 were compared with the first 45 patients treated with FLCC between April and June 2024. Allocation was based on chronological device availability. All procedures were performed by operators experienced in single-shot catheter ablation for AF. Patients were recruited from routine clinical practice at the Kerckhoff Heart Center, presenting with an indication for AF ablation. Exclusion criteria included the presence of intracavitary thrombus, severe valvular heart disease, or pregnancy.

PVI was performed in all patients using either VLCC or the FLCC PFA system. In selected cases, particularly in patients with persistent AF, enlarged left atria, or low-voltage substrate, additional ablation beyond PVI (eg, roof line or posterior wall isolation [PWI]) was performed at the operator’s discretion. In cases of FLCC, ablation beyond PVI, such as roof line or PWI, was performed only in patients with persistent AF and significant LA dilation, as identified by imaging, without the use of 3D mapping or low-voltage substrate information. Patients with concomitant typical atrial flutter underwent CTI ablation using the respective PFA system.

The study was approved by the Ethics Committee of Justus Liebig University Giessen (AZ 83/24) and conducted according to the Declaration of Helsinki. All patients gave written informed consent, including for potential ablation beyond PVI.

### Preprocedural management

In all patients, intracardiac thrombus was ruled out by transesophageal echocardiography prior to ablation. Additionally, transthoracic echocardiography was performed before the procedure to assess the LA area and left ventricular ejection fraction (LVEF).

In patients receiving novel oral anticoagulants (NOACs), the medication was paused for at least 12 hours before ablation. In contrast, patients on vitamin K antagonists continued their therapy with a target international normalized ratio between 2.0 and 3.0.

### Ablation procedure

All ablation procedures were performed under either deep sedation or general anesthesia by 4 experienced primary operators.

A 10-pole catheter was positioned in the coronary sinus for diagnostic purposes. Following a single transseptal puncture using the Brockenbrough technique (BRK-1) and an SL-1 sheath (Abbott, St. Paul, MN) intravenous heparin was administered to maintain an activated clotting time above 350 seconds. An exchange wire was positioned in the left superior PV (LSPV), allowing the transseptal sheath to be exchanged for a 10 F steerable sheath (FlexCath Contour™, Medtronic), which was used for both PFA systems. PV angiography was subsequently carried out to visualize the LA and PV anatomy. The respective PFA system was then introduced into the left atrium via the steerable sheath.

The VARIPULSE catheter (VLCC; Biosense Webster, Inc) is an open-irrigated 8.5 F ablation catheter equipped with 10 electrode rings. It features bidirectional steering with a D-curve allowing deflection in one direction and in the other, enabling precise engagement with all PVs The catheter’s distal loop has an adjustable diameter between 25 and 35 mm, allowing tailored conformation to individual vein anatomy for optimal tissue contact.^4^^,^^5^

After introduction of the VLCC into the left atrium, 3D electroanatomic mapping was initiated using the CARTO 3 system (version 7; Biosense Webster), and electrical signals within each PV were recorded. Following electroanatomical mapping, PFA was performed according to established protocols, consisting of at least 2 ostial and 2 antral applications per vein, resulting in a total of at least 16 PFA deliveries per patient prior to remapping. Each application comprised 3 sequences of bipolar energy delivery at 1800 V. Electrode-tissue contact was monitored via impedance-based visualization.^8^

Ablation sites were manually annotated on the electroanatomical map to track lesion locations. Remapping was conducted in all cases to verify durable PVI, and additional PFA applications were delivered if persistent conduction between the left atrium and PVs was observed after the initial lesions.

Further ablations, such as PWI or LA roof ablation (LARA), were performed at the discretion of the operator, for example, in patients with persistent AF and low-voltage areas. In accordance with the manufacturer’s recommendations, the total number of PFA applications using the VLCC system was limited to a maximum of 28 deliveries per procedure in the left atrium.

For low-voltage regions on the posterior wall, additional lesions were applied to create a posterior “box lesion”.

In patients undergoing additional LARA following PVI, the PFA catheter was placed in the LSPV and overlapping applications were delivered sequentially along the LA roof toward the right superior PV (RSPV), with gradual catheter repositioning via sheath retraction and rotation of PFA catheter.^9^ LA roof block was confirmed using differential pacing.

To evaluate bidirectional roof block, the PFA catheter was positioned at cranial and caudal regions of the posterior LA wall. Pacing from the right atrial septum (cycle length 500 ms) was used to assess activation times. LA roof block was indicated by a caudocranial activation sequence and a conduction delay >150 ms adjacent to the ablation line.^9^^,^^10^

In patients with concomitant typical atrial flutter, a 20-pole diagnostic catheter was positioned along the tricuspid annulus within the coronary sinus. Prior to CTI ablation, all patients received 0.1 mg of intravenous nitroglycerin to minimize the risk of coronary artery spasm. The CTI line was created by delivering overlapping pulsed field applications to achieve linear block; similarly, mitral isthmus line ablations were performed using the same approach. Following each energy application, a 12-lead electrocardiogram (ECG) was reviewed and compared to the baseline tracing to identify any signs of conduction abnormalities or ST-segment changes. Bidirectional block was then confirmed by pacing from both the lateral and septal sides of the ablation line.

In cases of SVC ablation, both entrance and exit block were assessed to confirm electrical isolation.

The procedural end point included both complete PVI (entrance and exit block) and confirmed conduction block across additional lines/lesions, assessed in sinus rhythm. All assessments were integrated into the routine procedural workflow without predefined observation periods. Complete 3D electroanatomic mapping of the left atrium and PVs was used before and after ablation in all VLCC cases the CARTO system.

In cases where the FLCC was used, the over-the-wire PulseSelect catheter was advanced into the left atrium via a steerable sheath and positioned within each PV to record electrical signals. Fluoroscopy was employed to confirm accurate catheter placement. The device features a 9 Fr shaft with bidirectional steering and a 25 mm circular loop comprising 9 electrodes capable of sensing, pacing, and delivering ablation.^3^ Per PV, at least 4 antral and 4 ostial applications were administered, each consisting of 4 biphasic, bipolar pulse trains. To ensure full PVI and address potential conduction gaps, particularly between electrodes 1 and 9, the catheter was rotated circumferentially into 4 distinct orientations following each application.

**Figure 1:**
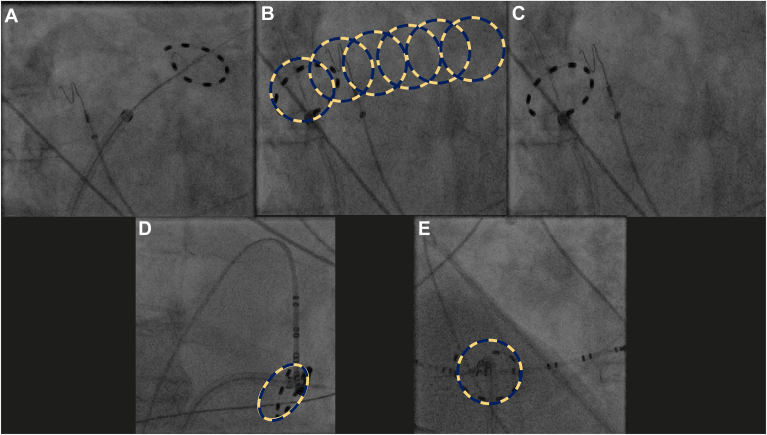
Maneuvers for roof line (A-C) and CTI ablation (D-E) using FLCC. **A:** First application (RAO). **B:** The roof line was created by slight sheath retraction and controlled rotation of the PFA catheter (RAO). **C:** Last application (RAO). **D:** CTI ablation (RAO). **E:** CTI ablation (LAO). CTI = cavotricuspid isthmus ablation; FLCC = fixed-loop circular catheter; LAO = left anterior oblique; RAO = right anterior oblique.

In cases of CTI and LARA ablation with FLCC, a comparable strategy was employed for the FLCC system as used with the VLCC (Figure 1). In contrast, procedures in the FLCC group were guided exclusively by fluoroscopy, without the use of a 3D electroanatomic mapping system.^6^

### Postprocedural management

A transthoracic echocardiography was performed immediately after the ablation to exclude pericardial effusion. Patients were continuously monitored via telemetry until discharge on the following day. Oral anticoagulation was resumed at least 3 hours post-procedure and continued for a minimum of 2 months, with further continuation based on the individual CHA_2_DS_2_-VA score. All patients received a proton pump inhibitor for 6 weeks. Antiarrhythmic medications were discontinued immediately after the ablation.

### End points

The primary efficacy end point was defined as acute procedural success, characterized by complete isolation of all PVs (entrance and exit block) as well as bidirectional conduction block across any additional linear lesions created during the procedure. Secondary end points encompassed procedural efficiency metrics, including total procedure duration, fluoroscopy time, and dose area product.

The primary safety end point was the incidence of predefined procedure-related or device-related complications, occurring intra-procedurally or during the index hospitalization.

For standardized comparison, procedures times, contrast medium usage and fluoroscopy time were assessed in the PVI-only group. Additional ablation strategies were reported descriptively.

## Statistical analysis

Continuous variables are reported as mean ± standard deviation or as median with interquartile range, depending on data distribution. Categorical variables are expressed as absolute numbers and percentages. Group comparisons were made using Student’s t-test or analysis of variance for continuous variables with a normal distribution, while the Mann–Whitney U test was applied for nonparametric data. For categorical variables, comparisons were performed using Pearson’s χ2 test or Fisher’s exact test, as appropriate. Statistical calculations were performed with the statistical analysis software R (R version 4.5.0 using RStudio IDE version 2025.05.1+513).

## Results

### Baseline characteristics

Baseline characteristics of the study population are summarized in Table 1. A total of 90 patients were included, with 45 undergoing PFA using the VLCC system and 45 with the FLCC system. Patient demographics and clinical profiles were largely comparable between the 2 groups. The proportion of female patients was similar (36% vs 29%, *P* = .652), as was the prevalence of persistent AF (51% vs 42%, *P* = .591). Mean body mass index (BMI) differed statistically (28.2 ± 4.3 vs 28.9 ± 4.0 kg/m^2^, *P* < .001), although the absolute difference was clinically negligible. Statistically significant differences were observed in median age (66 [IQR: 61–74] vs 64 [IQR: 55–74] years, *P* < .001), CHA_2_DS_2_-VA scores (2 [IQR: 1–3] in both groups, *P* < .001), and duration since first AF diagnosis (21 [IQR: 8–62] vs 12 [IQR: 4–40] months, *P* < .001). Comorbidities were similarly distributed between groups, with no statistically significant differences. Use of previous antiarrhythmic drugs was comparable across groups.

**Table 1:** Baseline characteristics

Parameters	VLCC N = 45	FLCC N = 45	*P*-value
Female gender, n	16 (36%)	13 (29%)	.652
Age, years	66 (61–74)	64 (55–74)	<.001
Persistent AF, n	23 (51%)	19 (42%)	.591
BMI, kg/m^2^	28.2 ± 4.3	28.9 ± 4.0	<.001
CHA_2_DS_2_-VA	2 (1–3)	2 (1–3)	<.001
Duration since first AF diagnosis, mos	21 (8–62)	12 (4–40)	<.001
Left atrial area, cm^2^	21.8 (17.5–24.1)	23.0 (18.2–27.0)	<.001
Left ventricular ejection fraction, %	60 (60–60)	60 (55–60)	<.001
Congestive heart failure, n	2 (4%)	4 (9%)	.677
Hypertension, n	34 (76%)	30 (67%)	.485
Diabetes mellitus, n	6 (13%)	7 (16%)	1.000
Obstructive sleep apnea, n	4 (9%)	5 (11%)	1.000
Coronary artery disease, n	8 (18%)	8 (18%)	1.000
Chronic obstructive pulmonary disease, n	1 (2%)	0 (0%)	1.000
Asthma, n	1 (2%)	1 (2%)	1.000
Pacemaker, n	0 (0%)	0 (0%)	NA
Implantable cardioverter defibrillator, n	0 (0%)	1 (2%)	1.000
History of stroke/Transient ischemic attack, n	3 (7%)	3 (7%)	1.000
Previous antiarrhythmic drugs			
Class I, n	4 (9%)	3 (7%)	1.000
Class II, n	38 (84%)	36 (80%)	.783
Class III, n	7 (16%)	10 (22%)	.590
Class IV, n	0 (0%)	0 (0%)	NA

Data are expressed as median (interquartile range), mean (standard deviation), or n (percentage).

AF = atrial fibrillation; BMI = body mass index; NA = not applicable.

Echocardiographic parameters, including LA area and LVEF, showed statistically significant differences but remained within a clinically comparable range (LA area: 21.8 [IQR: 17.5–24.1] vs 23.0 [IQR: 18.2–27.0] cm^2^, LVEF: 60 [IQR: 60–60]% vs 60 [IQR: 55–60]%, *P* < .001 for both).

### Procedural data

General anesthesia was not used in any VLCC cases, whereas it was administered in 9% of FLCC procedures (*P* = .116). Prior AF ablations were uncommon in both groups (2 patients in the VLCC group vs none in the FLCC group; *P* = .495).

Procedural data are shown in Table 2. Acute PVI was achieved in 100% of PVs using both VLCC (177/177) and FLCC (178/178) catheters. In the VLCC group, 3 patients had a left common ostium (LCO), while 2 patients in the FLCC group had an LCO. The median number of applications performed per patient was higher with FLCC (34 [IQR: 33–37]) compared to VLCC (18 [IQR: 17–19]).

**Table 2:** Procedural data - PV and non-PV sites

Parameters	VLCC N = 45	FLCC N = 45
Pulmonary vein (PV) isolation		
Total number of isolated PVs, n	177/177 (100%)	178/178 (100%)
Number of applications per patient, n	18 (17–19)	34 (33–37)
Left atrial roof ablation (LARA)		
Successful LARA, n	3/3 (100%)	12/12 (100%)
Number of applications per patient, n	4 (3–6)	7.5 (6–8)
Posterior wall isolation (PWI)		
Successful PWI, n	10 (100%)	1 (100%)
Number of applications per patient, n	6 (5–8)	12
Mitral isthmus line (MIL) ablation		
Successful MIL ablation, n	1/1 (100%)	-
Number of applications per patient, n	7	-
Superior vena cava (SVC) isolation		
Successful SVC Isolation, n	2/2 (100%)	-
Number of applications, n	3 (3–3)	-
Cavotricuspid isthmus (CTI) ablation		
Successful CTI ablation, n	4/4 (100%)	2/2 (100%)
Number of applications per patient, n	4 (3–5)	9 (5–13)
**RF touch-up needed, n**	0 (0%)	0 (0%)

Data are expressed as median (interquartile range), or n (percentage). For comparisons involving only 2 cases, values are given as minimum and maximum.

PV = pulmonary vein; RF = radiofrequency.

**Figure 2:**
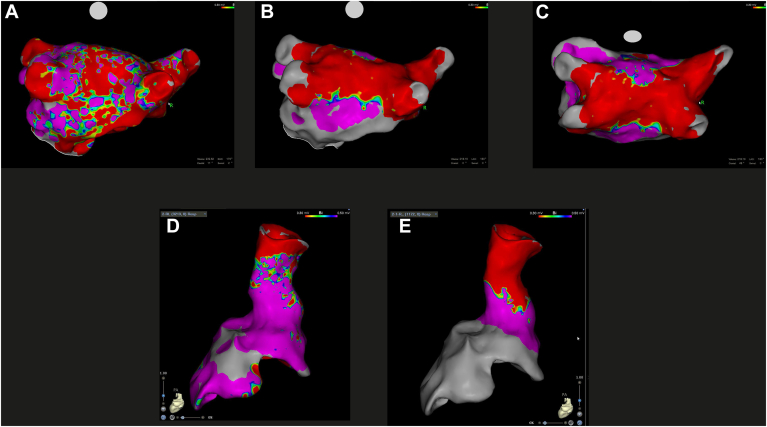
Electroanatomic map with variable-loop circular catheter. Left atrium and pulmonary veins in atrial fibrillation before **(A)** and after **(B and C)** pulmonary vein isolation and posterior wall isolation in sinus rhythm. Superior vena cava before (**D**) and after (**E**) isolation, both in sinus rhythm.

**Figure 3:**
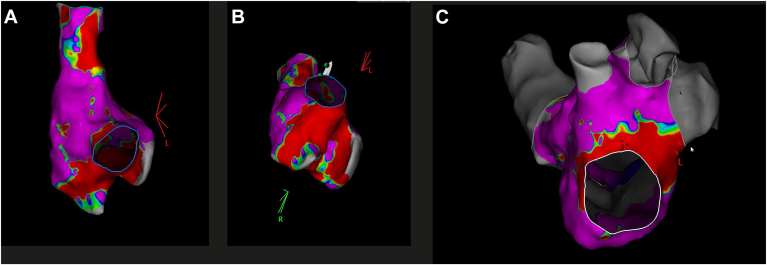
Electroanatomic map with variable-loop circular catheter. **A+ B:** Right atrium after cavotricuspid isthmus ablation. **C:** Left atrium after posterior mitral isthmus line ablation.

Additional ablation beyond PVI was performed in selected patients (Figures 2 and 3). LARA was successfully completed in 3 patients using VLCC and in 12 patients using FLCC, with a median of 4 (IQR: 3–6) and 7.5 (IQR: 6–8) applications per patient, respectively. PWI was performed in 10 patients in the VLCC group, with a median of 6 (IQR: 5–8) applications, and in 1 patient in the FLCC group with 12 applications. In the VLCC group, successful mitral isthmus line ablation was achieved in 1 patient (7 applications), and SVC isolation in 2 patients (3 applications each). CTI ablation was successfully performed in 4 VLCC patients (4 [IQR: 3–5]) and in 2 FLCC patients (median 9 [min: 5, max: 13]).

In some patients, additional ablation procedures were performed in combination during the same session. Among the 10 patients who underwent PWI with VLCC, 2 also received SVC isolation during the same procedure, and 1 patient additionally underwent CTI ablation.

All cases were first-time AF ablations except for 2 in the VLCC group. The first patient underwent a redo procedure with re-isolation of the right PVs, PWI, and SVC isolation. The second patient, undergoing a re-redo ablation, had re-isolation of the LCO and PWI prior to SVC isolation.

No additional radiofrequency (RF) touch-up applications were required in either group.

**Figure 4:**
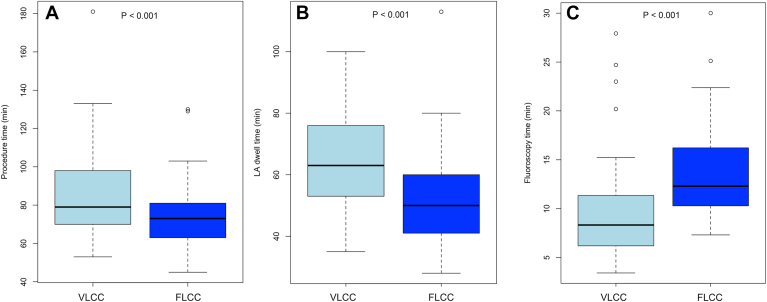
Comparison of procedural parameters between 2 circular pulsed field ablation catheters. A: Procedural time. B: Left atrial dwell time. C: Fluoroscopy time. FLCC = fixed-loop circular catheter. VLCC = variable-loop circular catheter.

Among patients undergoing PVI-only, significant differences were observed in procedural characteristics between the 2 catheter systems (Figure 4). Median total procedural time (skin-to-skin) was significantly longer in the VLCC group compared to the FLCC group (76.0 [IQR: 69.0–82.5] min vs 67.2 [IQR: 59.5–80.0] min, *P* < .001). Similarly, LA dwell time was longer in the VLCC group (58.0 [IQR: 52.8–66.0] min vs 47.0 [IQR: 38.0–58.5] min, *P* < .001). In contrast, median fluoroscopy time was significantly shorter in the VLCC group (8.3 [IQR: 5.9–11.4] min vs 11.4 [IQR: 10.1–14.0] min, *P* < .001), as was the total fluoroscopic dose area product (3.4 [IQR: 1.9–4.6] Gycm^2^ vs 5.7 [IQR: 3.3–7.8] Gycm^2^, *P* < .001). Median contrast medium volume was slightly lower in the VLCC group (17.0 [IQR: 15.0–20.0] mL vs 18.0 [IQR: 15.2–23.5] mL, *P* < .001), although the clinical relevance of this difference may be limited.

During the 1-day hospital stay, AF recurrence was observed in 1 patient from the VLCC group who had undergone combined PVI and CTI ablation. The patient was up-titrated on amiodarone, followed by successful electrical cardioversion.

### Complications

The complication rate did not differ significantly between the 2 groups. Major and minor complications are summarized in Table 3. In the FLCC group, 1 of 45 patients experienced a minor complication, while 3 complications occurred in the VLCC group (1 major and 2 minor; *P* = .620). Thus, the major adverse event rate was 2.2% in the VLCC group compared to 0% in the FLCC group (*P* = 1.000).

**Table 3:** Adverse events

Adverse events	FLCC N = 45	VLCC N = 45	*P*-value
Major adverse events			
Pericardial tamponade, n	0	0	-
Coronary artery spasm, n	0	0	-
Stroke, n	0	1 (2%)	1.000
Sustained phrenic nerve palsy, n	0	0	-
Atrioesophageal fistula, n	0	0	-
Thermal esophaegeal injury, n	0	0	-
Pulmonary vein stenosis, n	0	0	-
Major bleeding requiring transfusion, n	0	0	-
Vascular access complications requiring intervention, n	0	0	-
Death, n	0	0	-
Minor adverse events			
Pericardial effusion (no requiring intervention), n	0	0	-
Transient ischemic attack, n	0	0	-
Transient phrenic nerve palsy, n	0	0	-
Vascular access complications (no requiring intervention), n	1 (2%)	1 (2%)	1.000
Transient ST elevation, n	0	1 (2%)	1.000

Data are expressed as n (percentage).

In the FLCC group, minor bleeding and hematoma at the puncture site were successfully managed with compression. In the VLCC group, 1 patient developed transient ST-segment elevation followed by an ischemic stroke with left-sided hemiparesis. The patient was transferred to a neurological clinic for further evaluation and treatment. Transient ST-segment elevation was observed in the inferior ECG leads (II, III, and aVF) shortly after transseptal puncture. Coronary angiography was not performed due to reversible ST-segment elevation. The stroke was likely caused by a thromboembolic event related to the transseptal access. Another patient developed an arteriovenous (AV) fistula, which was successfully managed with manual compression.

All complications occurred in the PVI-only subgroup, except for 1 minor vascular event in the PVI-plus PWI subgroup. No other major complications (eg, pericardial effusion, esophageal injury, major bleeding, or death) were observed.

## Discussion

### Main findings

This study represents the first real-world comparison of the 2 circular PFA catheter systems, VARIPULSE and PulseSelect, during their clinical implementation. It further describes the use of these novel single-shot PFA devices for ablation beyond PVI in a high-volume center, demonstrating rapid feasibility, acute efficacy, and comparable safety for PVI and extended ablation strategies. While fluoroscopy time was higher in the FLCC group, overall procedural time was shorter compared to VLCC. Importantly, this study reports the first-in-man documented cases of CTI, mitral isthmus line ablation, and SVC isolation using the VLCC.

### Acute efficacy

Both the VLCC and FLCC systems demonstrated excellent acute efficacy, achieving 100% PVI without the need for RF touch-up in this real-world cohort. These results are in line with previously reported findings from the inspIRE, admIRE trials, and PULSED AF pivotal trial, which also demonstrated high acute PVI success with PFA in patients with AF.^3^, ^4^, ^5^ However, it is noteworthy that pivotal trials for the VLCC system primarily focused on paroxysmal AF, whereas in our cohort, a substantial proportion of patients (51%) presented with persistent AF, which further underscores the acute efficacy of the system in this more challenging patient group. The observed difference in the median number of PFA applications between FLCC (n = 34) and VLCC (n = 18) reflects the respective manufacturer-recommended ablation protocols, which specify a minimum number of applications per vein and loop configuration.

While previous reports have primarily focused on a pentaspline PFA catheter, demonstrating feasibility of ablation of non-PV sites such as CTI, mitral isthmus, and PWI ablation,^11^, ^12^, ^13^, ^14^ data on circular PFA catheters remain limited. In the admIRE trial, PWI was performed in 16 patients.^4^ However, real-world data on ablation beyond PVI with circular PFA systems, especially with VLCC, are sparse.

In this study, both PFA platforms enabled effective delivery of all additional lesion sets, including roof line, PWI, and CTI ablation, with high acute success rates and no need for RF touch-up.

CTI ablation has been described with high acute efficacy using various PFA systems.^15^ Our group previously reported successful CTI and roof line ablation using the FLCC platform.^6^ To our knowledge, ablation of right atrial structures, such as CTI and the SVC, has not yet been reported using VLCC.

We now report the first documented cases of CTI, mitral isthmus line, and SVC ablation using the VLCC system, with consistent success. These findings suggest a broader potential for VLCC in complex atrial substrate modification, particularly in redo AF procedures, compared to PFA systems lacking 3D mapping integration. Importantly, this is also the first report to describe the use of VLCC in redo ablations, an area where RF energy remains the predominant modality. By demonstrating successful re-isolation of PVs and additional substrate ablation using VLCC alone, this study supports the potential for workflow optimization in repeat procedures, potentially reducing overall procedure time while enhancing efficacy and safety.

Notably, fewer PFA applications were performed for these extended lesion sets in the VLCC group compared to FLCC group. This difference may be attributed to several factors: the variable-loop diameter of the VLCC catheter (25–35 mm) compared to the fixed 25-mm diameter of the FLCC system, as well as the integration of 3D electroanatomical mapping in VLCC cases. The use of 3D mapping likely enhanced visualization and anatomical precision during lesion deployment, in contrast to fluoroscopy-only guidance used in the FLCC group. It is important to note that while the FLCC catheter is not natively integrated into a 3D mapping system, it is compatible with such platforms. Thus, visualization of the lesion sets, and anatomical guidance would be feasible when used in conjunction with electroanatomical mapping.^16^

### Procedural times and fluoroscopy

Procedural differences between the VLCC and FLCC systems reflect inherent distinctions in catheter design and recommended workflows. In our cohort, VLCC procedures, performed with integration into 3D electroanatomical mapping, were associated with longer total procedure and LA dwell times, likely reflecting the additional steps required for 3D navigation, lesion annotation, and substrate visualization (76.0 vs 67.2 min; 58.0 vs 47.0 min, *P* < .001). In contrast, FLCC procedures, conducted under fluoroscopic guidance alone, were shorter in duration but involved higher radiation exposure (8.3 vs 11.4 min, *P* < .001).

Importantly, the use of VLCC with integration into 3D was associated with significantly reduced fluoroscopy time and dose, suggesting a procedural safety advantage for both patients and operators. The relatively long fluoroscopy time in the VLCC group, despite the use of an electroanatomical mapping system, can be explained by the fact that procedures were performed during the early clinical implementation phase of this novel technology. As such, operators initially relied more heavily on fluoroscopy for catheter positioning and safety confirmation. However, our results regarding procedural and fluoroscopy times are consistent with those reported in the inspIRE and admIRE trial.^4^^,^^5^ This consistency supports the generalizability of our findings, and reflects the expected procedural performance of both PFA systems during early clinical implementation.

Recent case series and the inspIRE and admIRE trials further suggest that fluoroscopy-free workflows may be feasible across all single-shot PFA systems when using 3D mapping or intracardiac echocardiography (ICE).^4^^,^^5^ This includes potential applications of FLCC under 3D mapping or ICE guidance, offering future pathways to reduce radiation exposure while maintaining procedural efficiency.^17^

### Safety

In this study, complication rates were comparable between the 2 PFA systems, VLCC and FLCC, underscoring their favorable safety profiles during clinical implementation. In the admIRE trial, the primary adverse event rate, comprising major complications, was 2.9%, while in the PULSED AF pivotal trial, it was 0.7%.^3^, ^4^, ^5^ These rates are comparable to our findings, with a major complication rate of 2.2% in the VLCC group and 0% in the FLCC group. Importantly, no cases of cardiac tamponade, esophageal injury, or procedure-related mortality were observed, particularly in the PVI-plus group, underscoring the strong safety profile of both PFA technologies. In our study, 1 patient in the VLCC group experienced an ischemic stroke following transient ST-segment elevation. Although initially attributed to transseptal puncture, a device-related embolic event cannot be excluded. It is important to note that embolic events, including ischemic strokes, have been reported with the VLCC system, and the manufacturer has publicly acknowledged such cases. This highlights the need for ongoing vigilance and further evaluation of PFA safety.

Ablation beyond the PVs has been increasingly recognized as safe and potentially beneficial in the treatment of persistent AF. Previous work from our group demonstrated that LARA performed with single-shot ablation systems (cryoballoon or PFA) in patients with persistent AF maintained a similar safety profile compared to PVI alone.^9^^,^^10^^,^^18^ PFA-based ablation beyond the PVs, such as PWI, has also shown an excellent safety record in multiple studies.^11^^,^^12^^,^^19^ While coronary artery spasms have been sporadically reported during mitral isthmus or CTI ablation using PFA systems, our study observed no such complications in either the VLCC or FLCC groups, likely due to the prophylactic administration of nitroglycerin prior to energy delivery.^1^

The findings suggest that VLCC and FLCC can be effectively integrated into early clinical practice for comprehensive AF ablation strategies, combining rapid procedural workflows with excellent safety.

### Clinical implications

This study confirms the feasibility and safety of both circular PFA systems for PVI and ablation beyond PVI in real-world clinical practice. By evaluating FLCC and VLCC during early implementation, including advanced lesion sets like roof line, CTI, and SVC isolation, the findings provide valuable insights into procedural workflows, efficiency, and fluoroscopy use. This can guide centers in selecting and integrating PFA technologies tailored to their needs. Notably, the integration of VLCC with 3D mapping offers potential advantages for complex and redo procedures, as illustrated by our initial clinical cases. However, further prospective studies are needed to assess long-term lesion durability and efficacy.

## Limitations

This study presents initial single-center experience with a limited cohort size and focuses on acute procedural outcomes. While structured follow-up beyond hospital discharge was not yet available, ongoing long-term data collection is underway to assess lesion durability and arrhythmia recurrence. These outcomes will be addressed in future analyses.

Another limitation of this study is the exclusive use of 3D mapping with the VLCC system, whereas FLCC procedures were performed without mapping support. This likely influenced procedural parameters such as fluoroscopy and LA dwell time. However, since both systems were applied according to their intended real-world workflows, with FLCC used for fluoroscopy-guided ablation and VLCC for 3D-guided ablation, this design reflects clinical practice and provides practical insight into system-specific advantages.

## Conclusion

This study presents the first clinical comparison of 2 circular PFA catheter systems for PVI and additional ablations beyond PVI. Both demonstrated high acute efficacy and favorable safety profiles. FLCC was linked to shorter procedural times, while VLCC reduced fluoroscopy exposure. Notably, this is the first report of successful CTI, SVC and mitral isthmus line ablation using VLCC. Further research is needed to assess long-term outcomes and lesion durability.

## Disclosures

JY, JH, PK, ML, MK, and TN received educational grants from Johnson & Johnson. MK reports advisory board activities for Medtronic and received fees from Medtronic. TN received fees from Johnson & Johnson. All other authors have reported that they have no relationships relevant to the contents of this paper to disclose.
